# Individual awareness and treatment effectiveness of hypertension among older adults in Ghana: evidence from the World Health Organization study of global ageing and adult health wave 2

**DOI:** 10.11604/pamj.2020.37.264.24526

**Published:** 2020-11-24

**Authors:** Benedict Calys-Tagoe, Benjamin D Nuertey, John Tetteh, Alfred Edwin Yawson

**Affiliations:** 1 Department of Community Health, University of Ghana Medical School, College of Health Sciences, University of Ghana, Accra, Ghana

**Keywords:** Self-reported hypertension, measured hypertension, WHO SAGE study, Ghana, older adults

## Abstract

**Introduction:**

the aim of this study was to report the prevalence of hypertension, its awareness and treatment effectiveness among older adults (aged 50 years and above) in Ghana.

**Methods:**

the Ghana World Health Organization´s (WHO) Study on Global Aging and Adult Health (SAGE) wave 2 dataset was used in this study. The study adopted a cross-sectional study design. Information on self-reported hypertension as well as measured hypertension was analyzed. The level of awareness regarding hypertension and the effectiveness of treatment was determined using descriptive statistics. Factors associated with an individual´s awareness of their hypertensive status were determined using Rao-Scott Chi square test statistic and the predictors of unawareness of hypertension were determined using adjusted logistic regression analysis. A p-value of ≤0.05 was deemed significant.

**Results:**

information on 3,575 adults in Ghana aged 50 years or older was included in this analysis. The mean age of study participants was 65.1 ± 10.7 years with 59% being female. The prevalence of measured hypertension was 50.7% [95%CI=48.3-53.2]. The overall prevalence of hypertension among older adults in Ghana who were hypertensive but were not aware of it was 35.0% [95%CI=31.6-38.5]. Of the 332 individuals who self-reported being hypertensive, only 74 (22.2%) were on any form of treatment, with only 17 (5.1%) having their blood pressures well controlled.

**Conclusion:**

approximately half of all older adults in Ghana have elevated blood pressures. Most of these are not aware of their elevated blood pressure and for those who are aware, very few are on treatment and even fewer have their blood pressure well controlled. Structured national population level screening and health promotion for elevated blood pressure by Ministry of Health/ Ghana Health Service is worthy of consideration.

## Introduction

Hypertension is the principal modifiable risk factor for cardiovascular diseases and it remains a major cause of health loss in all regions of the world [1, 2]. The global burden of diseases-ranked hypertension and associated cardiovascular diseases as the largest contributor to the global burden of diseases [3, 4]. Globally, high blood pressure went from being the fourth leading risk factor in 1990 to being the number one risk factor by 2010 [5, 6]. Almost a third of the world´s adult population have hypertension, accounting for over 1.39 billion individuals globally [7]. Hypertension accounts for 54% of strokes, 47% cases of ischaemic heart disease, 10.5 million deaths per year, 13.5% of all deaths worldwide and 92 million disability-adjusted life-years [8-10]. It is estimated that, 80% of global cardiovascular diseases burden and 86% of all strokes, occur in middle and low income countries [11, 12], suggestive of global hypertension disparities.

Hypertension is remarkably common in Africa and the prevalence is strongly influenced by age. Systematic reviews put the prevalence of hypertension among the older adults in Africa between 55.2% and 57.0% [13-16]. The prevalence of hypertension is increasing substantially, particularly in sub-Saharan Africa [4, 17]. Between the years 2000 to 2010, the age-standardized prevalence of hypertension increased by 7.7% in low and middle income countries [7]. In Ghana, hypertension, is ranked among the top five causes of morbidities, with a reported prevalence in the range of 19- 48% [18, 19] and self-reported hypertension prevalence of 15.8%. It is found to be more common in the elderly, [20,21] where prevalence has been reported in the range of 48-54% [22, 23]. Hypertension affects all socioeconomic classes, [11], however, there are significant disparities across various socioeconomic classes that affect the prevalence of awareness, treatment and control of hypertension in the elderly. A significant proportion of elderly with hypertension in communities remains undiagnosed, untreated or inadequately treated in sub Saharan Africa [17, 24]. Approximately 40% of all cardiovascular accidents occur in previously undiagnosed hypertensives [25]. Socio-demographic factors such as lower educational level, age and sex have been found to be associated with untreated hypertension and older age was significantly associated with uncontrolled hypertension [26].

Blood pressure is normally distributed in the population and there is no natural cut-off point above which hypertension definitively exists and below which it does not, however [27], epidemiological data have shown that increasing blood pressure has adverse effect on the risk of cardiovascular diseases including stroke, myocardial infarction, heart failure, peripheral artery disease and end-stage renal disease [28]. The risk associated with increasing blood pressure is continuous, with each 2mmHg rise in systolic blood pressure associated with a 7% increased risk of mortality from ischemic heart disease and a 10% increased risk of mortality from stroke [29]. These effects are even more pronounced in the elderly [26]. It is therefore important to identify individuals with high blood pressure in Ghana, increase uptake of hypertension treatment, and ensure that treatment for hypertensives are effective. The aim of this study is to report the prevalence of hypertension, its awareness, proportion on treatment and treatment effectiveness among older adults aged 50 years and above in Ghana. This study uses data from the World Health organization´s (WHO) Study on Global Aging and Adult Health (SAGE) wave 2 [30] to identify factors that are associated with unawareness (previously undiagnosed) and uncontrolled hypertension among this population so as to improve public health targeted interventions to address the high burden of unawareness, and uncontrolled hypertension in the elderly.

## Methods

**Study design:** SAGE wave 2 adopted a cross-sectional study design with a multi-stage cluster sampling technique. Details about the study design and procedures for data collection have been published elsewhere [31-33].

**Data source:** data from Wave 2 of the World Health Organization´s (WHO) study on global AGEing and adult health (SAGE) conducted between 2014-2015 in Ghana was used in this analysis. The SAGE study is a nationally representative, multi-country longitudinal study conducted among six countries that collects data to complement existing ageing data sources to inform health policy and programmes. WHO and the University of Ghana Medical School through the Department of Community Health collaborated to implement SAGE Wave 2. Detailed description of the methods used in the survey is published elsewhere [34].

**Study population:** individuals aged 50 years or older and a smaller sample of individuals aged 18-49 years were interviewed regarding their health care utilization, preventive health behaviours, chronic health conditions and health services coverage, subjective wellbeing and quality of life, risk factors and perceived health status, socio-demographic and work history, social cohesion and household characteristics. This study was based on adults aged =50 years. Further details about SAGE, especially about wave 2 can be found through the WHO website.

**Participants´ selection:** SAGE wave 2 sampling strategy was designed to account for expected attrition from wave 1 households (HH) which were visited for wave 2 data collection and replacements for sample attrition used a systematic sampling approach to randomly select new households. Mutually exclusive HH was classified into; SAGE wave 1 follow-up households with one or more members aged 50 years or older targeted for selection, new households with one or more members aged 50 years or older, SAGE wave 1 follow-up households which include residents aged 18-49 targeted for selection, new households which include residents aged 18-49 [32].

### Dependent variables and analysis

In this analysis, three categorical variables on hypertension were considered as dependent variables, these were; self-reported hypertension, measured hypertension and “unaware hypertension” (individuals found to have elevated blood pressure but were not aware prior to the study). Self-reported hypertension was captured in SAGE Wave 2 as a “Yes” response to the question: “Since we last spoke, have you been told by a doctor or health care professional that you have high blood pressure (hypertension)”. Measured hypertension was generated from anthropometric measurements involving three consecutive measured systolic and diastolic blood pressures. However, for our analysis, the arithmetic mean of the last two measurements for each individual was considered. Mean blood pressure of 140mmHg/90mmHg or higher was considered as “hypertension”. Unaware hypertension was generated from respondent who answered “No” to the question; “Since we last spoke, have you been told by a doctor or health care professional that you have high blood pressure (hypertension)”, but had their measured blood pressure ≥ 140/90mmHg. “Controlled hypertension” was defined as “blood pressure below 140/90mmHg in individuals being managed for hypertension [35]. Based on the STROBE (Strengthening the Reporting of Observational Studies in Epidemiology) recommendation for cross-sectional study design, implementation and reporting, missing response were strictly excluded in our analysis. There were 2,359 responses for self-reported hypertension and 3,528 for measured hypertension. For “unaware hypertension”, sample size was restricted to participants who self-reported hypertension as “No” (2027).

This study adjusted for the design nature of SAGE wave 2 (the clustering, stratification and individual weights). Descriptive statistics involved weighted row percentages involving cross-tabulation of independent variables associated with the dependent variables. This was done with a design-based chi-square value taking into consideration the design nature of SAGE Wave 2. Prevalence of self-reported and measured hypertension was calculated for both Wave 1 and Wave 2 of the SAGE study and these were compared. Scatter plot was used to demonstrate the clustering of systolic and diastolic BP among those who self-reported hypertension. Inferential statistics involving the use of binary adjusted logistic regression model was also carried out to assess the predictors of unaware hypertension. Stata 15 statistical software was used to perform the analysis.

**Ethical requirements:** the SAGE survey was approved by the World Health Organization's Ethical Review Board (reference number RPC149) and the University of Ghana College of Health Sciences, Ethical and Protocol Review Committee. Written informed consent was obtained from all study respondents.

## Results

**Figure 1:**
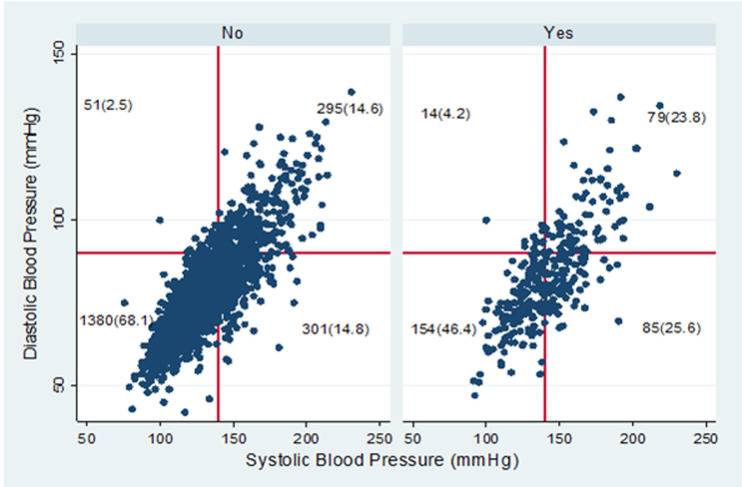
systolic and diastolic evaluation by status of self-reported hypertension among older adults in Ghana

**Figure 2:**
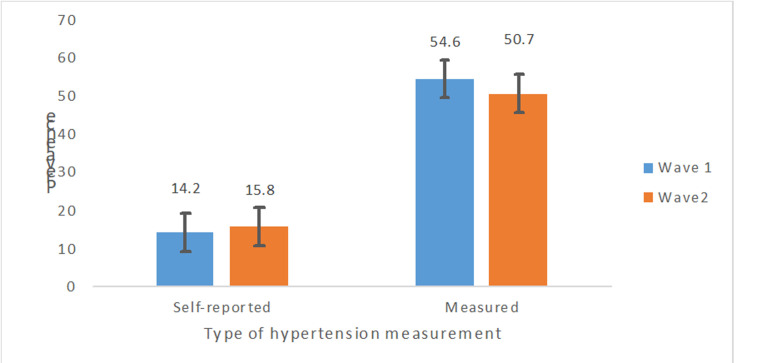
difference patterns of self-reported and measured hypertension among older adults between SAGE wave 1 wave 2

Information on 3,575 adults in Ghana aged 50 years or older was included in this analysis. Majority of them were females (58.8%), were currently married (56%), had some form of formal education (50.1%) and were Christians (71.7%). Their mean age was 65.1 ± 10.7 years. The prevalence of measured hypertension was 50.7% [95%CI=48.3-53.2]. Demographic and selected health-related characteristics of the older adults are as shown in Table 1. Rao-Scott chi-square test of independence showed a significant proportions of measured hypertension among age group, marital status, religion, place of residence, currently working, region, self-reported health, BMI and subjective well-being (p-value< 0.05) (Table 1). There was a positive linear relationship between systolic and diastolic BP among self-reported hypertensives as shown in Figure 1. Nearly a third (31.9%) of older adults in Ghana, who self-reported not having hypertension, had their measured BP = 140/90mmHg. Comparatively, about 53.6% of those who self-reported having hypertension still had BP=140/90 mmHg (Figure 1). However, the composite measured hypertension was 50.7% as presented in Figure 2.

**Table 1:** demographic characteristics associated with self-reported and measured hypertension among older adults in Ghana

Demography	Measured hypertension		Rao-Scott χ2(p-value)
Prevalence	50.7[48.3-53.2]	Total	
	Weighted %	N	
**Sex**			**0.11(0.74)**
Male	50.4	1461	
Female	51.1	2065	
**Age**			**11.89(<0.001)**
50-59	44.7	1275	
60-69	56.4	1090	
70-79	55.2	763	
80+	59.7	398	
**Marital status**			**10.36(<0.001)**
Never married	41.2	114	
Married	47.3	1980	
Separated/Divorced	54.4	419	
Widowed	59.5	1013	
**Religion**			**0.98(0.40)**
None	49.6	114	
Christian	51.3	2532	
Islam	50.9	658	
Primal indigenous	42.9	222	
**Place of residence**			**36.61(<0.001)**
Urban	58.4	1369	
Rural	43.6	2157	
**Currently working**			**38.06(<0.001)**
Yes	46.1	227	
No	60.4	1196	
**Region**			**9.38(<0.001)**
Ashanti	53.5	575	
Brong Ahafo	40.8	379	
Central	45	457	
Eastern	50.9	279	
GT. Accra	74.2	325	
Northern	45.5	356	
Upper East	36.7	196	
Upper West	40.5	185	
Volt	51.2	327	
Western	41.4	447	
**Self-reported health**			**3.77(0.007)**
Very good	50.9	222	
Good	46.9	1920	
Moderate	55.6	1025	
Bad	57.3	298	
Very bad	64.8	53	
**BMI**			**10.65(<0.001)**
Underweight	43.1	428	
Normal	46	1851	
Overweight	57.7	677	
Obesity	60.6	395	
**Subjective well-being**			**5.68(<0.001)**
Poor	62.8	228	
Low	54.7	1156	
Moderate	46.6	1855	
High	53.2	287	

**Figure 3:**
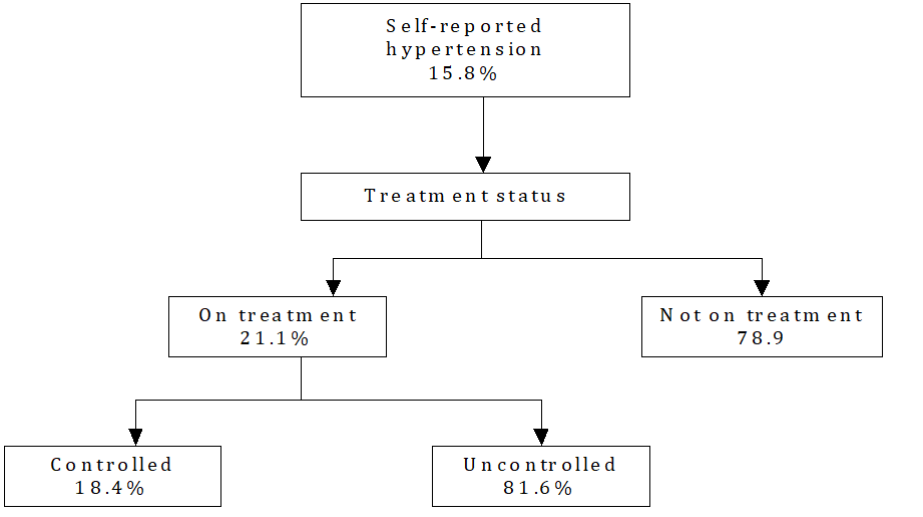
flow chart showing controlled and uncontrolled hypertension among self-reported hypertensive older adults in Ghana

The overall prevalence of older adults in Ghana who were hypertensive but were not aware of it was 35.0% [95%CI=31.6-38.5]. The variation of this prevalence rate with demographic and some selected health-related factors are shown in Table 2. Place of residence, current working status and subjective well-being were factors found to be associated with an individual´s level of awareness about their hypertensive status. Those living in urban locations, not working and reporting poor level of subjective well-being were significantly more likely to be unaware of their hypertension status (Table 2). Of the 332 individuals who self-reported being hypertensive, only 74 (21.1%) were on any form of treatment, with only 17 (18.4%) having their blood pressures well controlled (Figure 3).

**Table 2:** prevalence and associated risk factors of “unaware hypertension” among older adults in Ghana

Demographic variable	Unaware BP (35.0%)	Total	Chi-square(p-value)
	%	n=2027	
**Sex**			3.46(0.06)
Male	32.0	1005	
Female	38.6	1022	
**Age**			0.71(0.50)
50-59	33.1	545	
60-69	35.2	680	
70-79	38.8	521	
80+	35.3	281	
**Marital status**			2.48(0.07)
Never married	33.6	66	
Married	32.6	1044	
Separated/Divorced	33.6	245	
Widowed	41.2	672	
**Religion**			1.07(0.36)
None	42.5	63	
Christian	35.7	1461	
Islam	32.9	374	
Primal indigenous	28.0	125	
**Place of residence**			9.18(<0.005)
Urban	40.6	748	
Rural	30.2	1279	
**Currently working**			12.68(<0.001)
Yes	30.4	1296	
No	44.6	688	
**Region**			5.83(<0.001)
Ashanti	33.9	330	
Brong Ahafo	25.6	215	
Central	29.2	286	
Eastern	40.7	168	
GT. Accra	56.8	168	
Northern	32.3	209	
Upper East	22.8	112	
Upper West	24.7	82	
Volta	37.1	182	
Western	24.1	275	
**Self-reported health**			0.72(0.56)
Very good	33.6	129	
Good	33.2	1058	
Moderate	38.2	609	
Bad	38.1	188	
Very bad	38.2	31	
**BMI**			0.86(0.46)
Underweight	29.4	272	
Normal	33.6	1119	
Overweight	37.7	334	
Obesity	36.7	185	
**Subjective well-being**			3.01(0.04)
Poor	47.2	169	
Low	38.6	685	
Moderate	32.1	1012	
High	30.7	161	

**NOTE:** Weighted results

Predictors of an older adult in Ghana not being aware of their hypertensive status include their current working status (those not working were more likely to be unaware) and the region of residence (Table 3).

**Table 3:** predictors of unaware hypertension among older adults in Ghana

Characteristics	Predictors	aOR	P-value	95% Conf. Interval	
Unaware hypertension				Lower	Upper
	Sex				
	Female	Ref			
	Male	1.12	0.59	0.74	1.69
	Age				
	50-59	Ref			
	60-69	0.91	0.64	0.62	1.34
	70-79	1.01	0.94	0.74	1.38
	80+	0.73	0.12	0.49	1.08
	Marital status				
	Married	Ref			
	Never married	1.23	0.59	0.58	2.60
	Separated/Divorced	1.04	0.86	0.68	1.58
	Widowed	1.36	0.13	0.91	2.02
	Religion				
	Islam	Ref			
	None	1.74	0.15	0.82	3.68
	Christian	1.08	0.76	0.65	1.82
	Primal indigenous	1.14	0.68	0.62	2.09
	Place of residence				
	Urban	1.29	0.11	0.95	1.77
	Rural	Ref			
	Currently working				
	Yes	Ref			
	No	1.55	0.01	1.10	2.19
	Region				
	Upper East	Ref			
	Ashanti	1.65	0.09	0.92	2.95
	Brong Ahafo	1.15	0.67	0.60	2.21
	Central	1.34	0.27	0.80	2.25
	Eastern	2.22	0.02	1.12	4.40
	GT. Accra	4.02	0.001	1.79	8.99
	Northern	1.54	0.08	0.96	2.47
	Upper West	1.25	0.31	0.81	1.95
	Volta	1.78	0.04	1.03	3.09
	Western	0.96	0.88	0.55	1.68
	Self-reported health				
	Very good	Ref			
	Good	0.92	0.77	0.53	1.61
	Moderate	0.92	0.80	0.47	1.77
	Bad	0.80	0.52	0.41	1.57
	Very bad	0.47	0.11	0.19	1.19
	BMI				
	Normal	Ref			
	Underweight	0.82	0.30	0.57	1.19
	Overweight	1.10	0.68	0.71	1.69
	Obesity	0.81	0.38	0.50	1.30
	Subjective well-being				
	Poor	Ref			
	Low	0.77	0.31	0.47	1.28
	Moderate	0.70	0.20	0.41	1.21
	High	0.54	0.07	0.27	1.05

## Discussion

In this study, we set out to report the prevalence of hypertension, its awareness, proportion of patients on treatment and treatment effectiveness among older adults aged 50 years and above in Ghana. Approximately 1 out of every 2 of all older adults surveyed in Ghana have high blood pressure. Systematic reviews put the prevalence of hypertension among the older adults in Africa between 55.2% and 57.0% [13-16]. This study found 50.7% of the older adults to be hypertensive. Most of the studies used 60 years as the cut-off age for inclusion as an older adult. In the WHO SAGE methodology, older adults were classified as adults aged 50 years and above. The relatively lower prevalence observed in this analysis could probably be due to the inclusion of adults 50-59 years. The prevalence of measured hypertension among those aged 60 years and above in this study ranged between 55% and 59% and is in congruence with results from the systematic reviews [13-16].

One third of all older adults with high blood pressure were not aware of their hypertensive status. The low level of awareness are consistent with what is observed in many low and middle income countries in Africa [16, 36-38]. The low prevalence of diagnosed hypertension has public health implications for the likely complication of elevated blood pressure among otherwise healthy older adults in the communities [39]. Majority of strokes occur in undiagnosed hypertensives. Approximately 40% of all cardiovascular accidents occur in previously undiagnosed hypertensives [25]. This high prevalence of undiagnosed hypertensives raises a serious and potential public health crisis that requires urgent attention. Creating structured opportunities for blood pressure measurements among community dwelling members should be pursued as a way out of this imminent public health disaster.

Intuitively, this analysis found out that older adult females were more likely to be aware of their hypertension status compared to their male counterparts. Uncomplicated hypertension is often without symptoms and hypertension is usually diagnosed as incidental finding during assessment for some other medical conditions. There are several opportunities within the health care continuum for women to get a blood pressure measurement such as during antenatal clinic, family planning services, labour and delivery. Previous studies have shown that, the frequency of blood pressure measurement is associated with awareness of hypertension status [39]. In addition, disparities in health seeking behavior of men and women position women for regular medical check-up which results in an increase likelihood of being aware of hypertension status [19]. This agrees with Courtenay´s theory of gender and health which explains male hegemony regarding fear and perceived vulnerabilities to health seeking [40]. Men would thus not readily seek health care especially with chronic and usually symptomless condition such as hypertension.

The sensitivity of self-reported hypertension (SRH) for picking up hypertensives is very low especially in Africa. The rule of halves in hypertension and recent systematic review has shown that only half of hypertensives know their hypertensive status and would be identified by self-reporting in epidemiological studies [41, 42]. In the SAGE Wave 2, SRH missed two thirds of all those with hypertension among the older adults; suggesting that SRH has significant limitations that may bias estimates of hypertension among studies which depend solely on SRH in similar settings. Between the six year interval of SAGE study measured hypertension decreased by 4% from 54.6% to 50.7% [43]. This marginal increase in awareness of hypertension status among older adults with hypertension was however not statistically significant. With the migration of Ghana from low to lower middle income status; higher educational and health infrastructure development within the six years interval may have contributed to this marginal, though not significant increase in level of awareness [44].

Prevalence of measured hypertension as well as that of self-reported hypertension are more common in urban than rural communities. This is consistent with findings in several part of Africa indicating hypertension to be more prevalent in urban communities. There is evidence that prevalence of hypertension among Ghanaians increases across the following; rural, urban, and Ghanaian migrant communities [45]. There is also evidence that the prevalence of awareness of hypertension status also increases across the rural-urban communities. In contrast, this study found a lower prevalence of awareness of hypertension status among the urban dwellers compared to rural dwellers. This observation requires further exploration and a concerted national effort to reach these older urban dwelling adults for hypertension diagnosis and management. Amazingly, nearly four out of every five (79%) older adults who self-reported being hypertensive, were not on any form of treatment. Again, less than one in every five (18%) older adults on treatment for hypertension had their blood pressure well controlled. Low compliance to treatment protocol by healthcare providers, increased defaulter rate, multiplicity of drug treatment regimen and lifestyle modifications not routinely provided to persons with hypertension have been associated with these observations. [46]. Increased defaulter rate occur from misconception about side effects of medications for managing hypertension, easy access to herbal medications marketed as cure for hypertension, cost of antihypertensive medications and inadequate adherence counselling [46]. In addition, treatment resistant hypertension have been associated with age, and some inhibitory patient factors [46].

The strength of this study is that, it used a nationally representative sample of older adults such that the findings are generalizable to the population of older adults in Ghana. It also used a robust methodology that has been tested and currently used by all six countries taking part in the WHO SAGE study. However, there were some limitations. A single day measurement of blood pressure was used in the determination of high blood pressure. However, three measurements were made separated by about 30 minute´s interval and the average of the last two measurements made was used to estimate the systolic and diastolic blood pressure.

## Conclusion

Hypertension is very prevalent among older adults in Ghana with one out of every two adults being affected. Many older adults in Ghana who have high blood pressure are unaware of their hypertensive status. Major factors predicting an individual´s unawareness of their hypertensive status include their current working status and their region of residence. For those who are aware of their hypertensive status, only a fifth are on treatment, with over 80% of those on treatment having poorly controlled blood pressures. Studies have shown that increased frequency of visits to hospital increase the likelihood of being aware of hypertension status. We recommend the following: public health interventions that allow community health nurses to check the blood pressure of the elderly during home visits. This would increase regular blood pressure measurement and increase the prevalence of awareness of hypertension among older adults; healthcare providers should be encouraged and challenged to offer adherence counselling to patients put on treatment for hypertension since it is a chronic condition and the treatment is usually over a prolong period, if not for life. This will enhance patients´ understanding of their condition as well as the treatment modalities and ultimately may improve compliance which is a major reason for poorly controlled hypertension; additionally, the health system should explore the use of communication techniques to improve medication adherence. The use of electronic health records which can be programmed to generate alerts about persons prescribed antihypertensive medications but failed to refill over time will be helpful in tracking defaulters; clinicians should adhere to clinical support decision tools to improve the care rendered to the elderly.

### What is known about this topic

- Hypertension is ranked among the top five causes of morbidities in Ghana;
- 15.8% self-reported hypertension exist among older adults in Ghana;
- Interventions to improve awareness and early detection of hypertension at population level is key.

### What this study adds

- Hypertension is ranked among the top five causes of morbidities in Ghana;
- 15.8% self-reported hypertension exist among older adults in Ghana;
- Interventions to improve awareness and early detection of hypertension at population level is key.
